# Gambling and Migration – The Role of Culture and Family

**DOI:** 10.1007/s10899-024-10292-9

**Published:** 2024-04-09

**Authors:** Anders Nilsson, Youstina Demetry, Shervin Shahnavaz, Johanna Gripenberg, Pia Kvillemo

**Affiliations:** Department of Clinical Neuroscience, Centre for Psychiatry Research, Karolinska Institutet, & Stockholm Health Care Services, Stockholm, Sweden

**Keywords:** Gambling, Problem gambling, Migration, Culture

## Abstract

Problem gambling (PG) is a public health concern with severe repercussions for the individual, concerned significant others and the society. Foreign borns generally gamble less but are overrepresented among those with PG. Previous research has suggested that other factors, such as socio-economic status, might explain this, but also that cultural factors might play a role in the relationship to gambling and the development of PG. This qualitative study using content analysis investigates the experiences of and opinions about gambling and PG among 12 males living in Sweden with a migrant background in Afghanistan, the Middle East and North Africa. The results were show that the acculturation process could be a factor in developing PG, as well as a cultural values regarding money and wealth. PG was seen as more stigmatized in the origin country, and the family played a more important role in the rehabilitation of PG compared to the general population. The results of this study could be used to inform preventive and clinical programs to better reach people with a migrant background.

## Introduction

Problem gambling (PG) is a public health concern affecting approximately 1–2% of the world population (Gabellini et al., 2023; Williams et al., 2012). PG is associated with a long range of harms for the individual with PG, as well as their concerned significant others and the community as a whole (Langham et al., 2016). PG is unevenly distributed in the population, with higher prevalence found among certain groups such as males, younger adults and groups with lower socio-economic status (Allami et al., 2021; Folkhälsomyndigheten, 2019). Several studies have also identified higher PG prevalence among ethnic minorities compared to majority population (Okuda et al., 2016; Wardle et al., 2019). The annual Swedish national public health survey estimated in 2021 that among those born outside of Europe, 10.5% of males and 3.5% of females had some degree of PG, compared to 4.6% of males and 1.4% of females born in Sweden (Folkhälsomyndigheten, 2021). Similar figures can be found in the longitudinal gambling study Swelogs where 5.7% of individuals born outside of Sweden have some degree of PG compared to 1.4% of those born in Sweden (Folkhälsomyndigheten, 2019). In neighboring Denmark, corresponding findings have been found for individuals born in “non-Western” countries compared to those born in Denmark (Kragelund et al., 2022). Approximately 11.7% of the Swedish population is born outside of Europe, making this a large, albeit heterogenous, group (Statistiska Centralbyrån, 2023).

For other psychiatric disorders, foreign borns in Europe tend to have a lower risk of developing risky alcohol use (Hjern & Allebeck, 2004), lower risk to commit suicide (Hollander et al., 2020), but a higher risk to develop psychosis and PTSD (Fox et al., 2021; Selten et al., 2020), and a similar risk as the general population of developing anxiety and depression disorders (Foo et al., 2018). There are also differences within this heterogenous group, where those with a refugee background seem to have a higher risk of psychiatric disorders compared to other immigrants (Tinghög et al., 2017).

Previous research has pointed to a paradoxical phenomenon where foreign borns tend to gamble less as a group, but have elevated levels of PG (Wardle et al., 2019). In a review article of 38 studies from eight “Western” countries, Wardle et al. (2019) identified three overarching themes of which two could increase the motivation to gamble and the risk of developing PG and one could protect against it. The first theme was problems with the acculturation process (i.e. the process of learning and incorporating the values, beliefs, language, etc. of the new country), such as isolation, unemployment, communication problems or boredom, which could be a risk factor for developing PG. The second theme was advertisement and availability of gambling in the new country, which could serve as a risk factor for PG, not least since it could be in stark contrast to the situation in the country of origin, where it could be more restricted or prohibited. The third theme, which could function as a protective factor against PG, was negative religious and moral beliefs and values regarding gambling. However, these results should be interpreted with caution, since they involve individuals with very heterogenous cultural and economic backgrounds, arriving in eight different countries which share some, but far from all, cultural, economic and legal systems.

There is no consensus on how to understand the higher prevalence of PG among minority groups (with or without a refugee background), but it has been suggested that it could be explained by factors such as socio-economic status, comorbid psychiatric disorders and difficulties relating to the integration process (Çakıcı et al., 2021; Jacoby et al., 2013; Raylu & Oei, 2004). Other studies have pointed to specific cultural customs and beliefs that could increase the risk of developing PG for certain groups, such as notions of fatality destiny and chance in Chinese culture (Chee & Lui, 2021; Papineau, 2001), and the importance of financial success and high social status among Americans with Iranian background (Parhami et al., 2012). Okuda et al. (2016) theorized that cultural differences in relationship to gambling and PG might be due to two different processes. First, different cultural groups might be exposed to different levels of risk factors related to gambling, such as perceived norms or availability of gambling. Second, culture might influence the susceptibility to those gambling risk factors, such as whether gambling risk factors are moderated by or interact with cultural factors.

In the Swedish and northern European setting, migrants born outside of Europe largely originate from the Middle East, North Africa and Asia (Statistiska Centralbyrån, 2023). A large proportion of immigrants have arrived as refugees from countries such as Iraq, Iran, Turkey, Syria, Somalia and Afghanistan. There is limited research on PG in these specific migrant groups, but one Australian study on an Arabic speaking helpline for PG highlighted the specific role Islam has, since it holds more negative views on gambling compared to other religions (Mazbouh-Moussa & Ohtsuka, 2017). This could be protective against developing PG since it might influence people to abstain from gambling (Mutti-Packer et al., 2017), but it might have a negative effect on help-seeking, since PG becomes highly stigmatized. In an Australian report on shame and stigma of PG, stigma is identified as an especially important problem among migrants from the Middle East with PG (Hing et al., 2015).

Despite the high prevalence of PG among people with a migrant background, little is known about gambling and PG in this group. The aim of this study is to investigate the experiences, attitudes and thoughts about gambling and PG among individuals with a migrant background from Afghanistan, Iran, Iraq, Tunisia and Turkey in Sweden.

### Research Questions

- What are the experiences of and thoughts about gambling and problem gambling among males from the Middle East, North Africa and Afghanistan?
- Have the participants experienced any differences between Sweden and the origin country in the perception of gambling and problem gambling?
- Have any cultural factors affected the participants’ relationship to gambling and the development of problem gambling?

## Methods

This qualitative study consists of interviews with 12 individuals with a migrant background who self-identified as gamblers or former gamblers. Background information of the informants is displayed in Table 1.

### Interviews

The research team developed a semi-structured interview guide based on previous research on PG. The interview questions concerned experiences of gambling, gambling among family and friends, thoughts on gambling in origin country as compared to Sweden and thoughts on help-seeking for PG, see Appendix 1. The interviewers had no prior relationship to either of the participants. AN and PK had previously conducted research and interviews on PG, albeit not related to migrant backgrounds, while YD had primarily conducted research on psychological treatments for other conditions, but targeting people with a migrant background. The interviewers were instructed to use probing statements and explore unexpected information from the informants too. The interviews were performed by the authors AN, YD or PK, either face-to-face, through video calls or via phone calls between December 2021 and October 2022. Of the participants, nine were in their homes when being interviewed, while three were interviewed in a public setting, but no other persons were present at the interview. All interviews were conducted in Swedish, although the research team had the possibility to interview the participants in Arabic by YD, Persian by SS and English by any of the interviewers, as well as with an interpreter for other languages. The interviews lasted between 20 and 60 min. The interviewers made notes during the interviews, but the interviews were also audio recorded and transcribed verbatim.

### Analysis

The interviews were analyzed using qualitative content analysis (Hsieh & Shannon, 2005). Qualitative content analysis is a flexible family of methods, covering impressionistic, intuitive, interpretive, as well as strict textual analytical approaches, with possibilities to handle manifest as well as latent content [10]. Content analysis can be used in a more inductive form (conventional), if theories on the research topic are lacking, as well as deductive form (directed) when theories to build on are present. The research team chose the conventional approach, as there is limited theoretical understanding of the studied phenomenon.

One of the authors (AN), read the transcribed interviews repeatedly, trying to get an overall impression of the material. During the reading, AN developed a preliminary coding scheme with definitions (codebook) (MacQueen et al., 1998). The scheme was presented to the other authors who came with suggestions of alterations. Subsequently, two of the authors (AN and PK) coded the material independent of each other, with a high degree of consensus. Throughout this process, the authors read and re-read the transcriptions to assure that the new codes adequately reflected the content in the transcriptions. The codes were then analyzed to identify possible themes and subthemes, which were subsequently organized in Table 2. AN and PK decided that data was saturated after the 12 interviews were conducted.

### Participants

The 12 participants were required to have experiences of gambling, albeit not necessarily PG, and were recruited through addiction clinics, peer support groups, and personal contacts. An additional four prospective participants showed an interest to participate, but were not reachable when the research team tried to contact them. The participants were all male, and had a migrant background from Afghanistan, Iran, Iraq, Tunisia or Turkey, see Table 1. All but one was born outside of Sweden, and all self-identified as gamblers or former gamblers. The prospective participants were informed about the general aim of the study; i.e., to investigate the experiences of and opinions about gambling and PG among people living in Sweden with a migrant background. The participants were informed that their participation was voluntary and that they could withdraw their consent to participate at any time. No monetary or other compensation was given for participating in the study.

**Table 1:** Participant characteristics

ID	Age	Age on time of arrival to Sweden	Origin country	Participated in treatment	Main problem game
ID1	59	22	Iraq	*	Casino, online casino
ID2	20	14	Afghanistan	Yes	Online casino, sports betting online
ID3	44	24	Turkey	Yes	Online casino, sports betting online
ID4	55	16	Turkey	Yes	Online casino
ID5	42	9	Iran	Yes	Poker
ID6	47	4	Turkey	Yes	Online casino, sports betting online, horse betting, poker
ID7	36	5	Turkey (Kurdistan)	No	Sports betting online
ID8	41	25	Tunisia	Yes	Online casino, casino
ID9	65	25	Iran	Yes	Online poker, online casino
ID10	37	Born in Sweden	Turkey	No	Sports betting online, online casino
ID11	42	16	Iran	Yes	Online casino
ID12	39	6	Iran	*	Poker, casino, online casino

* Participant was not asked about participation in treatment or support for PG

## Results

The results were divided into 5 themes and 15 subthemes, see Table 2. These represent different aspects of the participants relationship to and perception of gambling and PG.

**Table 2:** *Themes and subthemes*

Theme	Relationship to gambling	Acculturation and gambling	Gambling in Sweden vs. origin country	Cultural views on gambling	Help-seeking and treatment
Subtheme	Problem gambling	Financial motives	Availability and regulation	Stigma and shame	Family-oriented problem solving
	Gambling in the family/ among friends	Alienation and belonging	Unpreparedness	Religious views	Unconditional family support
	Peer support groups	Sudden access to money	Everyday gambling	Extravagance and status	Professional treatment and support

### Relationship to Gambling

The first theme covers the general relationship to gambling for the participants, and has three subthemes.

#### Problem Gambling

Most participants self-described as being or having been “problem gamblers”, with periods of uncontrolled gambling and often comprehensive negative consequences in terms of economy and relationships, and in one instance eventually leading to a prison sentence:

*“I used every mean I could think of: gambling, drugs, sex and alcohol. I either play nothing or I take it very far”.* ID12.

Some participants asserted that they did not have a gambling problem, but would admit to experiencing negative consequences from it:

*“Sometimes I think it’s too bad if I’ve gambled for 10,000 [Swedish crowns = 900 EUR]. If I lose 100,000 [9000 EUR] in a year, I think about how I could have given it to someone poor, to charity.”* ID7.

#### Gambling in the Family/Among Friends

A common theme was that the participants had been exposed to gambling from older family members when growing up:

*“My dad and my uncles, they would play cards in the evenings. I was three or four years old and I would sometimes sit in my dad’s lap and watch them play. It wasn’t always about money, but I could see the joy in their eyes when they won and how they talked to each other and the body language which was very fascinating. And when we got older, we, the cousins, sat and played the same games.”* ID5.

Some would also mention gambling among friends when growing up:

*“First time [I gambled] was when I was 12, in Iran. It was betting with friends, different types of sports. It was a kick to keep up the interest for the sport, but it was also social”.* ID11.

#### Peer Support Groups

Some participants had engaged in various peer support groups for PG:

*“I have suffered a lot and that’s why I’ve become active and engaged in a peer support organization”.* ID9.

### Acculturation and Gambling

The second theme encompasses subthemes covering gambling that can be related to the process of migration and integration.

#### Financial Motives

Gambling had in some instances been partly driven by financial motives that were linked to the migration and integration in Sweden:

*“Many [migrants] have a difficult time economically, and they also have a responsibility to provide for several people in the home country”.* ID 11.

#### Alienation and Belonging

Gambling seemed for some to be an activity that offered a place of belonging when otherwise experiencing alienation from society:

*“We [migrants] are very detached from a lot of things in Sweden. Detached from society. (---) So, we either become addicted to alcohol or gambling”.* ID3.

*“I think that the process of becoming a part of Sweden [is an explanation]. You don’t have any social network and gambling becomes a hobby. Something social”.* ID11.

#### Sudden Access to Money

To some, coming to Sweden eventually signified an elevated economic status, that would prompt them to use large amounts of money on gambling:

*“We made of lot of money from our businesses at that time. There was a lot of money and we had an abundance of money, we didn’t know what to do with it.”* ID6.

### Gambling in Sweden vs. Origin Country

There were several marked differences in relation to gambling between Sweden and the origin countries.

#### Availability and Regulation

In all origin countries except for Turkey, gambling was illegal and the availability was experienced as low:

*“When I was in my home country, we had no…there were no casinos or anything. Nor any possibility to gamble online”.* ID8.

#### Unpreparedness

To some participants, gambling was a novel phenomenon when arriving in Sweden:

*“Before coming here, I didn’t know about roulette and such things. I learned about roulette and lotto and then things got worse and worse”.* ID1.

#### Everyday Gambling

Several participants reported that certain types of everyday gambling was extensive and widespread in their origin countries and cultures:

*” It’s so integrated in our countries that no matter what you do, you bet on it. And that’s a form of gambling, gambling isn’t just roulette, black jack, poker or online casinos”.* ID5.

*” To be honest, when we were kids and played out in the yard and played ludo or card games, we always played for something. There was always something we played for. It was either comic books or ice cream. If we played football, the winners were given ice cream by the opponents. It was always something”.* ID6.

### Cultural Views on Gambling

This theme covers perceptions of gambling that differ between the origin culture and Swedish culture.

#### Shame and Stigma

The participants unanimously highlighted that gambling and PG was highly stigmatized in their origin cultures:

*“It’s pretty harsh when it comes to gambling, since our culture or religion doesn’t allow you to gamble”.* ID10.

*“I don’t want to tell my relatives in my home country, it’s nothing to be proud of. They don’t understand that it is a type of disease.”* ID8.

#### Religious Views

A few of the participants expressed that religion (Islam) played an important role in how gambling was viewed in their origin country, albeit not necessarily for themselves:

*“It (gambling) is also a sin, it’s haram. So, it’s considered more shameful there”.* ID7.

#### Extravagance and Status

Gambling was in some instances seen as a way to display monetary wealth and success:

*“One thing that is cultural, all over the Middle East, is that it’s a “society of prestige” where being rich has a very high status. It’s obvious at the casino that there is a cultural connection: most people are either East Asians or from the Middle East. That’s why we buy expensive things, gamble big etc. The image you project towards your friends and the rest of society is very important in our cultures”.* ID12.

### Help-Seeking and Treatment

This theme covers aspects of the participants experiences of and opinions about treatment seeking and support, both for themselves and for others with a similar background.

#### Family-oriented Problem Solving

Several participants underscored the importance of family in providing support for people with PG:

*“Immigrants in general don’t want to admit (to having PG) to the same extent (as native Swedes). It doesn’t matter what type of dependency; you want to fix it within the family. You don’t want to let it be known to outsiders that you have these problems*”. ID5.

While some underlined the importance for many to not let problems be known outside the immediate family, others pointed to support being given by a larger part of the family:

*“I have a cousin (---), and that idiot had gambled away his wife’s gold. He was pretty young back then, like 20–25. (---) They were just about to buy a new house, his parents that is. So, this blew up. So, all the cousins and everyone who could helped out. And I sent 20 000–30 000, and my mom also sent like 20–30. And all the cousins in Europe who could help to get them back on their feet. But he wasn’t very popular then. And there was a bit of a fuzz, but I didn’t want to have anything back”. ID7*.

#### Unconditional Support

Similar to the above subtheme, some participants expressed that the family support for someone who had some form of dependency was unconditional, which sometimes clashed with principles taught by treatment providers in Sweden:

*“When it comes to Swedish culture, and I notice that from the psychologists and therapists too, they’ll for example say: “if you see him do this or that, kick him out. Kick him out, he’s on his own”. In our culture, it’s not so damn easy to kick out your child onto the street. No matter if he’s a drug addict, alcoholic or gambling addict”.* ID6.

#### Professional Support and Treatment

Most participants had experience from professional treatment or support for PG, which was generally seen as a positive experience:

*“I thought it was good. It helps. I’ve gone to treatment now and I haven’t played for two months. I’ve accepted my debts and I’ve stopped dreaming about the big win. And I’ve learned how to handle cravings”.* ID8.

But the participants that had not received any treatment expressed some skepticism about its value:

*“No, I wouldn’t contact health care, really. But I think I’d start with my friends. And I would try to get a sense of how bad it was, how bad I felt”.* ID10.

## Discussion

This study provides some important insights into the role of culture and migration for gambling and PG. The participants had all experienced negative consequences from gambling and often self-described as ‘problem gamblers’. The results suggest that experiences related to migration, integration and culture could have an important role in shaping the relationship to gambling, and to the development of PG. The results can largely be understood as being in line with the two-process model proposed by Okuda et al. (2016), where culture affects both which risk factors are present, but also the susceptibility for those risk factors. In this study, we suggest that the exposure to everyday gambling at young age is an example of the first, and the stigma surrounding PG an example of the latter.

The participants differed in age of migration and during which time period they migrated to Sweden. However, it was clear that experiences that could be related to migration play an important role in their relationship to gambling, as accounted for in the Acculturation and gambling theme. This included gambling as a response to experiences of economic hardship due to the migration, language barriers, alienation or lack of a sense of coherence, gambling as a way of passing time and coping pressing life circumstances, unpreparedness for gambling availability and advertisements which seem to confirm previous findings (Wardle et al., 2019). In some instances, it also entailed an unexpected and markedly higher and economic status that could trigger gambling. It should be stressed, however, that the participants also highlighted processes and factors unrelated to migration and culture in relation to gambling and the development of PG, such as the games’ inherently reinforcing properties and the availability of gambling at venues serving alcohol.

The perhaps most salient result from this study was how the participants considered gambling, and PG, to be substantially more stigmatized in the origin countries compared to in Sweden, described under the Shame and stigma subtheme. The participants talk about gambling being seen as “a disgrace”, “not something to be talked about” or “haram”, referencing a term in Islamic nomenclature denouncing something as forbidden or unlawful. These norms regarding gambling are also mirrored in the legal framework of the origin countries of the participants, where gambling is forbidden in all countries except Turkey, where some lotteries are allowed (DLA Piper, 2022). This could also be an explanation for why some of the participants claimed to have very limited prior knowledge about gambling before coming to Sweden, and could also increase vulnerability to gambling advertising. The stigma surrounding gambling and PG is seen as a barrier to seek treatment, which corroborates claims from numerous previous studies (Gainsbury et al., 2014; Suurvali et al., 2009), and seem to prompt individuals with PG to rather handle the problems by themselves or within the family. Several participants also highlighted the important role the family and relatives had in supporting someone with PG, in contrast to more individualistic practices in Sweden, where family members are encouraged by counselors to avoid paying for gambling-related expenses for example. The participants generally had a favorable view of treatment for PG, but asserted that ‘others’ with similar cultural background as themselves would generally be reluctant to seek formal treatment or support, which has been found in previous studies of groups with a similar background (Mazbouh-Moussa & Ohtsuka, 2017). A recent study from Norway indicated that people with a migrant background were more likely to have PG but less likely to seek treatment for it, but with time and after obtaining citizenship they became more likely to seek treatment for PG (Aarestad et al., 2023). This is also similar to previous studies on treatment seeking for mental problems among populations with a migrant background that have found lower levels of treatment seeking relative to the rest of the population, despite similar levels of mental health problems (Hollander et al., 2020). This has partly been explained by differences in ‘health literacy’ i.e., the capacity for an individual to receive and understand fundamental health information and make informed decisions (Nutbeam, 2008), but also by a lack of culturally attuned treatment options (Wong et al., 2015).

Despite the marked stigma surrounding gambling, several participants described gambling as an integral part of everyday life in their origin countries and cultures. This type of gambling, however, is different from the type of organized and commercial gambling we usually refer to when talking about gambling. Instead, this type of gambling was described as betting between friends about everyday events, card games and dice games involving small sums of money or tokens such as chewing gums or candies. Different cultural contexts will influence how this type of gambling is perceived, as described by Egerer and Marionneau (2019). In their study French and Finnish gamblers had markedly different views on what was described as ‘convenience gambling’, which, albeit commercial, is reminiscent of the type of gambling described by the respondents in our study. However, since early exposure to, and participation in, gambling has been found to be a possible risk factor for developing PG (Freund et al., 2022), this type of gambling could have a role in the development of PG for some individuals. In a recent study of PG among minority groups (Grant & Chamberlain, 2023), using pooled data from ten different studies on PG, the age of first gambling was significantly lower among treatment-seeking individuals from minority groups (age 14 on average) compared to non-minority treatment-seekers (age 22 on average), indicating that early onset gambling could be an important factor explaining some of the difference in PG prevalence.

One finding from this study suggested that cultural norms encouraging aspirations for a high and highly visible economic status could be important reasons to gamble. This was also linked to a certain way of gambling, where extravagance and fearlessness could be preferred over more restrictive and cunning styles of play, despite worse outcomes. This echoes some previous findings among Americans with Iranian background, where culturally driven desires to achieve and maintain high economic status has been proposed to partly explain elevated PG prevalence figures (Parhami et al., 2012). For some participants, earning money to send to family members in the origin country had also been a reason to gamble. Some of these findings could be interpreted as a part of a specifically masculine context, since all of the participants were male. Gambling and PG is generally more common among males, and masculine norms regarding risk taking, competition, status and help-seeking could have an influence on gambling and the development of PG (Hunt & Gonsalkorale, 2018). On a population level, female migrants are more likely to migrate as relatives, thus arriving to an existing familial network, while male migrants are more likely to be refugees or migrant laborers without existing networks and contacts, which could be a risk factor for gambling (Allami et al., 2021).

This study is one of the first studies on the experiences of gambling and PG among people with a migrant background, despite approximately 14.6% of the US population being foreign-born (United States Census Bureau, 2021) and 8.4% of the EU population being born outside the EU (European Comission, 2021), for example. However, the sheer number of individuals this entails also calls for some caution when interpreting the results of this and other studies on migration background; the participants’ culture, socio-economic status, education level and reasons to migrate might differ substantially and contribute in different ways. It is also important to stress that the term ‘culture’ can sometimes be misleading, since it might indicate that customs, traditions, beliefs and values in a certain community, country or region are static or easily described, whereas in reality ‘culture’ is an ongoing process that can vary substantially between time periods, location and how it is experienced and perceived. It is also important not to overstate the role of culture, when other aspects such as socio-economic status or masculinity might be equally, or more, important.

In sum, cultural factors and experiences from the migration and integration process can be understood to have played an important role in the participants’ relationship to gambling and development of PG. Stigma seemed to be an important barrier for seeking treatment, which together with a culturally ill-adapted health care system might deter certain groups to seek treatment. This study highlights the need to reconsider how treatments are offered, where an overly narrow focus on the individual might clash with culturally rooted understandings of PG as a problem to solve within or with the support of the family. Previous studies lend preliminary support for the viability of culturally adapted PG treatment programs for minorities (Bertossa, 2022; Richard et al., 2017; Wong et al., 2015). Other studies have for example investigated the different views and experiences held by older generations compared to the rest of the population (Heiskanen & Matilainen, 2020), highlighting the importance of an intersectional approach that take into account different demographic, cultural or social backgrounds when preventing and treating PG.

In conclusion, this study lends some support to the notion that cultural practices and experiences specific to migrants could influence the risk of developing PG. Given the elevated prevalence of PG among many minorities, and the challenge to reach and provide relevant prevention and treatment programs for these groups, there is an urgent need to better understand cultural aspects in relation to gambling and PG. The results from this study could help inform prevention programs on relevant and culturally attuned themes regarding for example stigma surrounding PG, the role of gambling to achieve higher social status and what role gambling can play in coping with adaptation and integration into a new society. But the experiences of the participants also highlighted the need to address the knowledge gap regarding the risks of gambling demonstrated by some migrants with little prior experience of gambling or gambling advertisements in their origin countries. Future studies should further investigate the role different specific cultural practices could have in shaping the relationship to gambling, which could help build a theoretical model for how to understand PG in minority groups.

### Limitations

Two of the largest migrant groups in Europe, Syrians and Somalis, are not represented in the study which limits what conclusion that can be drawn. The participants were also all male, and it is very likely that a more even gender distribution would have provided different results. It should be noted that a majority of the participants had received some type of professional support or treatment for PG. Though this is not a limitation per se, it is likely to have an important influence on their views on gambling, PG and treatment for PG and should be taken into account when interpreting the results.

## Data Availability

Data will be made available upon reasonable request.
